# Experimental study of PLLA/INH slow release implant fabricated by three dimensional printing technique and drug release characteristics in vitro

**DOI:** 10.1186/1475-925X-13-97

**Published:** 2014-07-19

**Authors:** Gui Wu, Weigang Wu, Qixin Zheng, Jingfeng Li, Jianbo Zhou, Zhilei Hu

**Affiliations:** 1 Department of Orthopaedics, Union Hospital, Tongji Medical College, Huazhong University of Science And Technology, Wuhan 430022, People’s Republic of China; 2 Department of orthopedics, 2nd Affiliated Hospital, School of Medicine, Zhejiang University, Hangzhou 310009, People’s Republic of China

**Keywords:** Three dimensional printing technique, Doughnut shape, Implant, PLLA

## Abstract

**Background:**

Local slow release implant provided long term and stable drug release in the lesion. The objective of this study was to fabricate biodegradable slow release INH/PLLA tablet via 3 dimensional printing technique (3DP) and to compare the drug release characteristics of three different structured tablets in vitro.

**Methods:**

Three different drug delivery systems (columnar-shaped tablet (CST), doughnut-shaped tablet (DST) and multilayer doughnut-shaped tablet (MDST)) were manufactured by the three dimensional printing machine and isoniazid was loaded into the implant. Dynamic soaking method was used to study the drug release characteristics of the three implants. MTT cytotoxicity test and direct contact test were utilized to study the biocompatibility of the implant. The microstructures of the implants’ surfaces were observed with electron microscope.

**Results:**

The PLLA powder in the tablet could be excellently combined through 3DP without disintegration. Electron microscope observations showed that INH distributed evenly on the surface of the tablet in a “nest-shaped” way, while the surface of the barrier layer in the multilayer doughnut shaped tablet was compact and did not contain INH. The concentration of INH in all of the three tablets were still higher than the effective bacteriostasis concentration (Isoniazid: 0.025 ~ 0.05 μg/ml) after 30 day’s release in vitro. All of the tablets showed initial burst release of the INH in the early period. Drug concentration of MDST became stable and had little fluctuation starting from the 6th day of the release. Drug concentration of DST and CST decreased gradually and the rate of decrease in concentration was faster in DST than CST. MTT cytotoxicity test and direct contact test indicated that the INH-PLLA tablet had low cytotoxicity and favorable biocompatibility.

**Conclusions:**

Three dimensional printing technique was a reliable technique to fabricate complicated implants. Drug release pattern in MDST was the most stable among the three implants. It was an ideal drug delivery system for the antibiotics. Biocompatibility tests demonstrated that the INH-PLLA implant did not have cytotoxicity. The multilayer donut-shaped tablet provided a new constant slow release method after an initial burst for the topical application of the antibiotic.

## Background

Local slow release implant provided long term and stable drug release in the lesion. In contrast to oral drug administration, it increased the topical drug concentration while the blood drug concentration was maintained at a relatively low level. In this way, local drug delivery enhanced the drug therapeutic efficacy and decreased systemic side effects. Recent local drug delivery systems were mainly used for treating tumor and chronic infectious diseases [1,2]. In order to manufacture drug delivery devices that could release drug smoothly, and for the drug release rate to reach or close to zero order, researchers studied various drug loading devices, including doughnut shaped tablets, multilayer tablets and microparticles etc. [3-5].

Doughnut shaped tablets maintained the stability of drug release rate through keeping the effective drug release surface of the device constant. Traditionally, such devices were produced through compressing the mixture of drug and the drug loading matrix. However, the uniformity of the mixture was limited. Detachment between the different layers of the multilayer tablet processed by compression was likely to happen. Those disadvantages directly influenced the drug release pattern.

The application of three dimensional printing (3DP) technique had received wide attention [6-8], since the technique was first introduced by Sachs etc. 3DP machine fabricated 3D structure models designed by computer, through binding powders layer by layer with binding liquid. It had great advantages in processing drug loading implants with complicated internal structures [9-11]. Model drugs could be dissolved in binding liquid evenly and injected on the powder layer by the printing head very precisely. The effective therapeutic concentration of the model drug should be low, due to the limitation of drug loading ability of the implant. INH as a classic antituberculosis agent with minimal inhibitory concentration between 0.025 ~ 0.05 μg/ml was an ideal model drug. In this study we manufactured an INH-PLLA implant via 3DP technique, compared the release feature of different structures of implants in vitro, and studied the biocompatibility of the INH-PLLA implants created via 3DP technique.

## Methods

### Material and equipment

PLLA, M_w_ = 100 KD, was provided by Dikan Biomedical Co., Ltd (Chengdu, China). Isoniazide was purchased from TianRui Pharmaceutical Co., Ltd (Zhejiang, China). Acetone and alcohol were obtained from Shi Yi Chemical Reagent Limited Company (Shanghai, China). 3 DP machine was designed and built by Fochif Mechatronics Technique Co. Ltd (Shanghai, China).

### Preparation of binding liquid and PLLA powder

Binding liquid was the mixture of acetone, alcohol and DI water at the ratio of 20:4:5 (v/v). Moreover, it also contained some accessory ingredients, including glycerol, polyvinylpyrrolidone and lauryl sodium sulfate. INH bulk drug was dissolved in the binding liquid at a concentration of 120 mg/ml. INH containing and absent binding liquid were filled into ink boxes respectively. Ground PLLA powder with a diameter between 50-75 um was selected after processing by a grinder. All the drugs and powders were stored in a P_2_O_5_ vacuum dryer.

### Structure design of the implant

**Figure 1:**
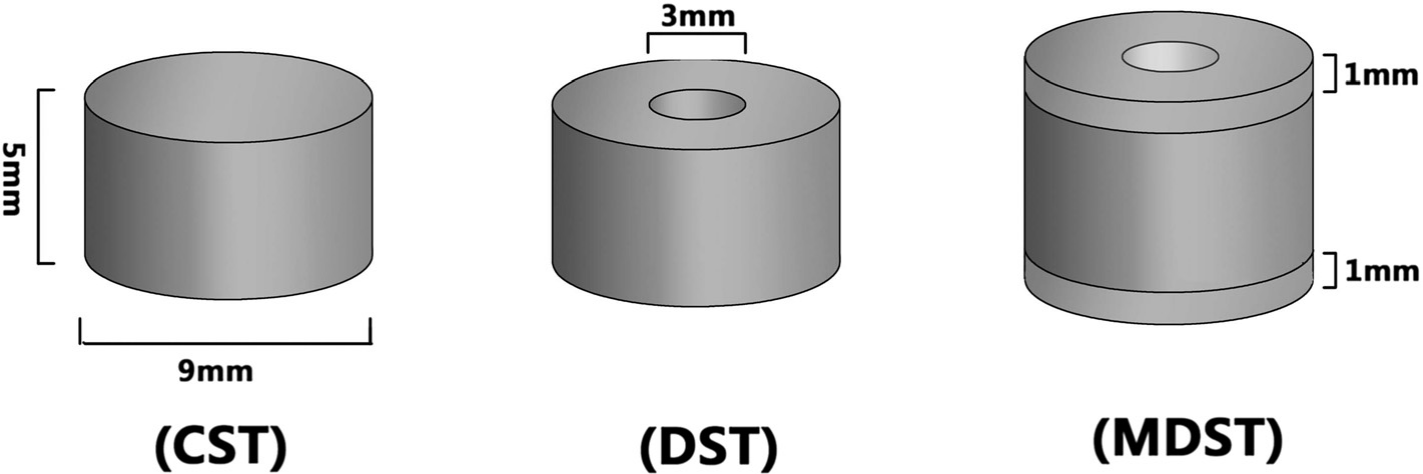
INH-PLLA implant ideograph.

In this research study we designed three implants with different structures, as shown in Figure 1. The structures were columnar-shaped tablet (CST), doughnut-shaped tablet (DST) and multilayer doughnut-shaped tabled (MDST), with an outer diameter of 9 mm. Their heights were 5 mm, 5 mm, 7 mm respectively. Both DST and MDST had a hole in the center with a diameter of 3 mm. MDST had barrier layers at the top and bottom extremities of the implant with a thickness of 1 mm each. The volume and the surface area of the implant was shown in Table 1.

**Table 1:** Common feature of the implants

**Dosage form**	**Drug loading volume (mm**^**3**^**)**	**Drug release surface (mm**^**2**^**)**
CST	317.9	268.5
DST	282.6	301.47
MDST	282.6	188.4

### Fabrication of the implant

**Figure 2:**
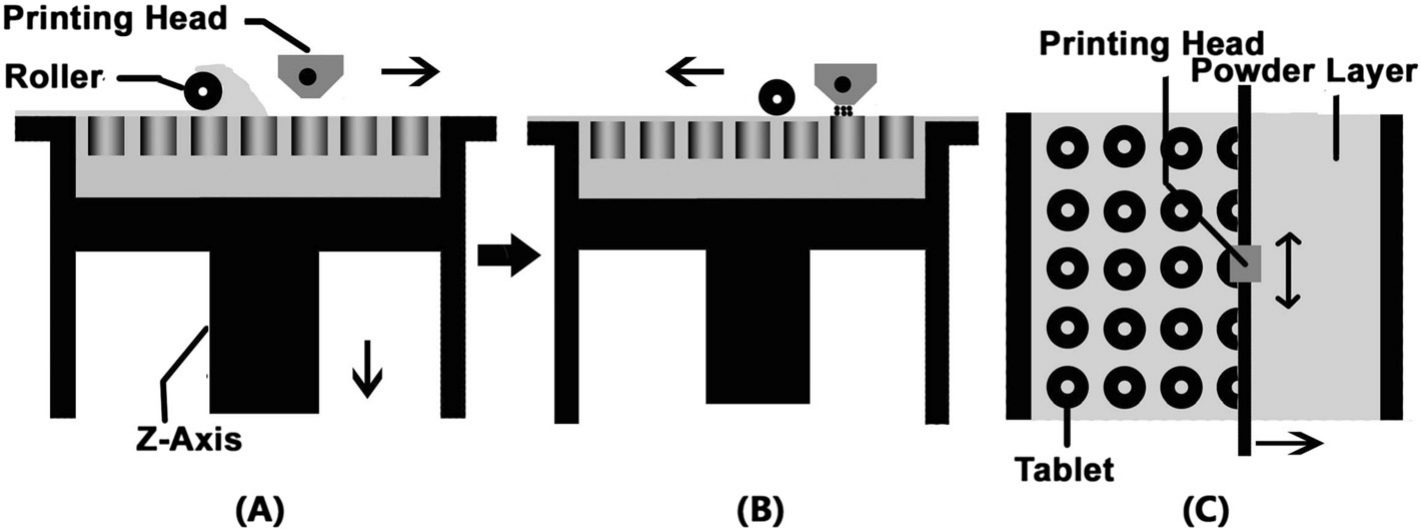
**Schematic diagram of the 3D printing. A**: Lateral view of the printing machine; The roller smooth the powder when it’s moving; **B**: Lateral view of the printing machine; The printing head inject the binding liquid on the suface of the powder layer; **C**: Anterior view of the printing machine; Several round circle patterns are printed on the PLLA powder layer.

The printing parameter was shown in Table 2. The machine was reset and covered with a layer of PLLA powder on the surface of the working platform. The printing machine would smooth the powder automatically with the roller, and then binding liquid would be injected on the surface of the PLLA powder layer to form a certain pattern according to the printing parameters. To combine the powder particles more firmly, injection of the binding liquid would be repeated as necessary. The working platform descended a certain height in the direction of z-axis when the combination of a single layer was finished. Another PLLA powder layer could be laid with the same thickness, and then the machine injected binding liquid at the same place as the previous layer. Thus the two PLLA powder layers combined together. Repeating those processes, the machine could fabricate certain shapes of PLLA implant according to the model programmed in the computer (Figure 2).

**Table 2:** Printing parameter of the INH-PLLA implant with different structures

**Pattern**	**Layer thickness/μm**	**Layer**	**Printing time**	**Binding liquid**
CST	200	1st-25th	15	INH-binding liquid
DST	200	1st-25th	15	INH-binding liquid
MDST	200	1st-5th & 31st-35th	15	Empty-binding liquid
	200	6th-30th	15	INH-binding liquid

After the printing process, the drug implants were kept inside the PLLA powder to dry for 30 min under room temperature. Additional PLLA powder was removed and the implants were stored in the P_2_O_5_ vacuum dryer. The PLLA powder on the surface was removed 12 hours later. To combine the powder particles more firmly, a few coats of binding liquid was sprinkled on the surface of the implant evenly. The barrier layers of the MDST were grinded on the surface of the glass which was moistened by acetone 7 days after the printing. All the products were dried for 12 days in the vacuum dryer.

### Biocompatibility detection

#### ***Separation and cultivation of the rat mesenchymal stem cell***

SD rats of either gender were decapitated and soaked in 70% alcohol for 10 min; the femur and tibia of both posterior legs of the rats were separated under aseptic technique and the soft tissue was removed with PBS solution; the bone was cut and the bone marrow was washed into the 10 ml aseptic centrifuge tube with 10 ml medium containing 10% FBS. The cell suspension was collected into 15 ml centrifuge tube and divided into two equal shares. The cell suspension was centrifuged for 15 min at the speed of 1000 rpm/min under room temperature. The supernatant was abandoned and 5 ml culture medium was added to suspend the cells again. The cell suspension was inoculated into 6-well plates and cultured at 37°C, 5% CO_2_ thermostat. Half of the culture medium was replaced 24 hours after the inoculation, and the fluid was thereafter changed every 3 days. The cells were sub-cultured after 80-90% confluence.

#### ***Preparation of leaching liquor***

100 mg of the INH-PLLA implant was lixiviated in 500 ml fetal bovine serum at 37°C attemperator for 3 days. Filter liquor was sterilized by filtrating through 0.22 um micropore film. The filter liquor was stored in the refrigerator at 4°C.

#### ***Cytotoxicity assay (MTT)***

Rat mesenchymal stem cell were digested by 0.25% trypsin to prepare cell suspension. The cell density was modulated to 2 × 10^4^/ml. 200 μl cell suspension was seeded into each well of 96-well culture plate and cultured in incubator at 37°C with 5% CO_2_ for 24 h to make the cells attached. 15 wells were set as experimental group and another 15 wells as control group. The original culture medium in each group was altered with 200 μl leaching liquor and normal DMEM culture medium respectively. The cells were cultured in the incubator at 37°C with 5% CO_2_. 5 wells of each group were selected after being cultured for 24 h, 48 h and 72 h. Then 20 μl MTT (5 mg/ml) was added and incubated for 4 h at 37°C. The supernatant was removed and DMSO 150 μl was added, and shaken for 10 min. The OD value was measured at immune microplate reader under 490 nm wavelenth. The relative growth rate (RGR) was caculated on the basis of the mean OD value of each timing. RGR% = 100 × OD_sample_/OD_control_.

#### ***Direct contact test***

The implants were sterilized with ethylene oxide and cut into small pieces with a size of about 2 × 2 × 2 mm. The rat mesenchymal stem cell suspension was kept agitated. 2 ml cell suspension was imbibedand seeded in the wells of 6-well culture plate. 3 wells were set as experimental group and the other 3 as control group. The small pieces of INH-PLLA implant were placed into the experimental group and kept the implant undisturbed. The cells were cultured in the incubator at 37°C with 5% CO_2_. After 1, 2 and 3 days, the inverted phase contrast microscope was used to observe the cell morphology and proliferation.

### In vitro drug release experiment

Soaking method was used in the in vitro release experiment. Deionized water was the release medium. 10 ml deionized water was filled into a glass bottle. The implant was tied up with a fine line and hung in the release medium. The glass bottle was kept in the constant temperature vibrator which vibrating rate was 100 times/min at 37°C. 10 ml solution was taken out every other day and stored under frozen condition, and then the bottle was filled with 10 ml fresh deionized water. The release experiment lasted 30 days.

The sample was tested with RT-HPLC. The ODS-SP (4.6 × 250 × 5u) was used as chromatographic column. The flowing phase was methanol-DSP (0.02 mol/L, the pH was adjusted to 4.5 with phosphoricacid) (75:25, V/V). The UV wave length was 254 nm and the flowing rate was 1 ml/min.

### Data statistical analysis

The experimental data was analyzed with SPSS13.0 software, and the result was expressed as $\bar{X}\pm S$. *T*-test was used for comparision between groups of MTT and P < 0.05 was statistically significant.

## Results

### Appearance of the INH-PLLA implant

**Figure 3:**
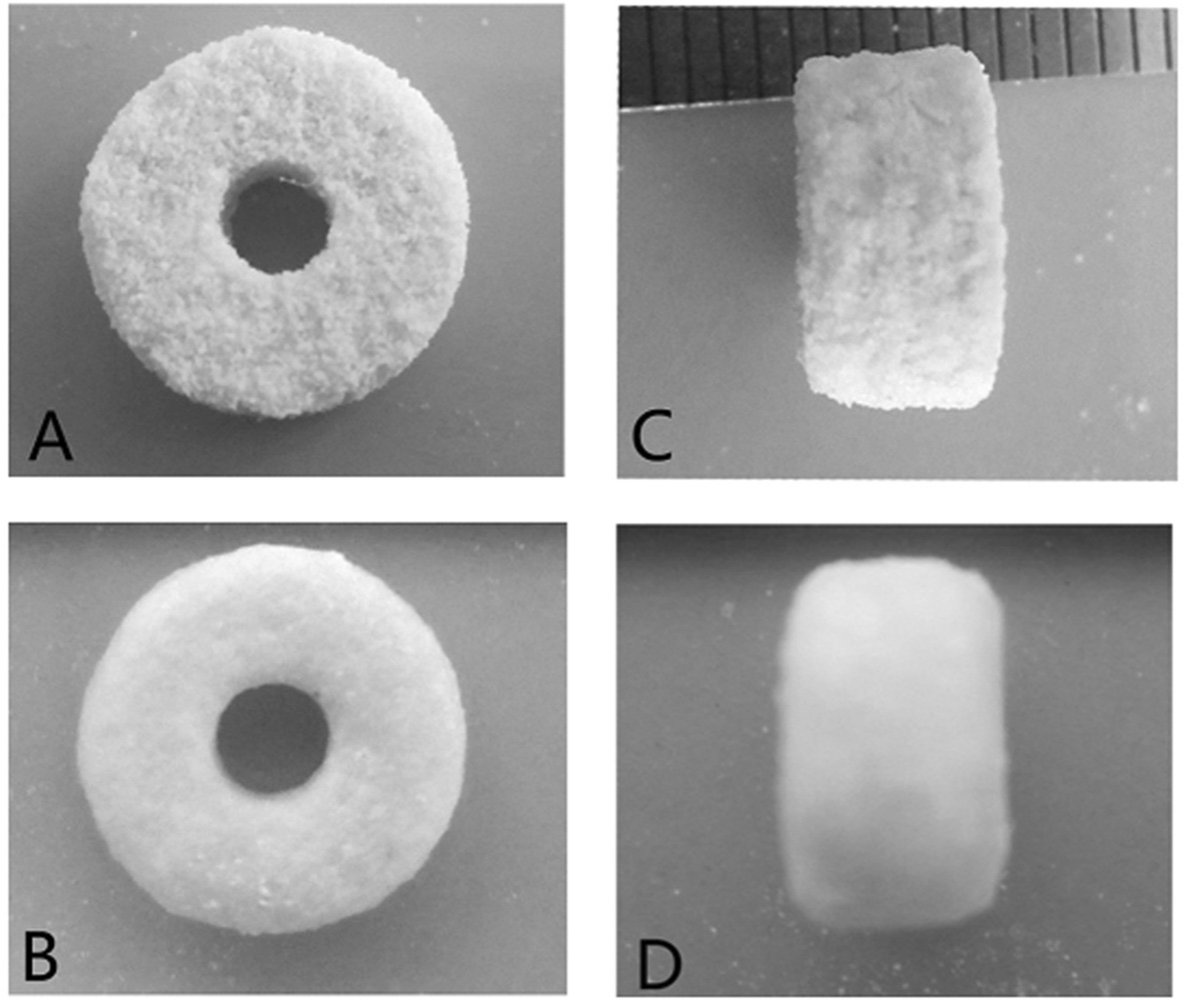
**Appearance of the implant. A**: Anterior view of the semi-finished implant; **B**: Anterior view of the finished implant; **C**: Lateral view of the semi-finished implant; **D**: Lateral view of the finished implant.

The PLLA implant is shown in Figure 3. The combination of the PLLA powder was quite compact and there was no separation between layers. The size of the implant corresponded to the pre-set parameters. However, granular particles were found on the surface of the semi-finished implant, and the particles may fall off from the surface of the implant. After the post printing processing, the particles bonded more closely and no additional powder fell off. The holes in the implant surface also reduced in size.

### Microstructure of the INH-PLLA implant

**Figure 4:**
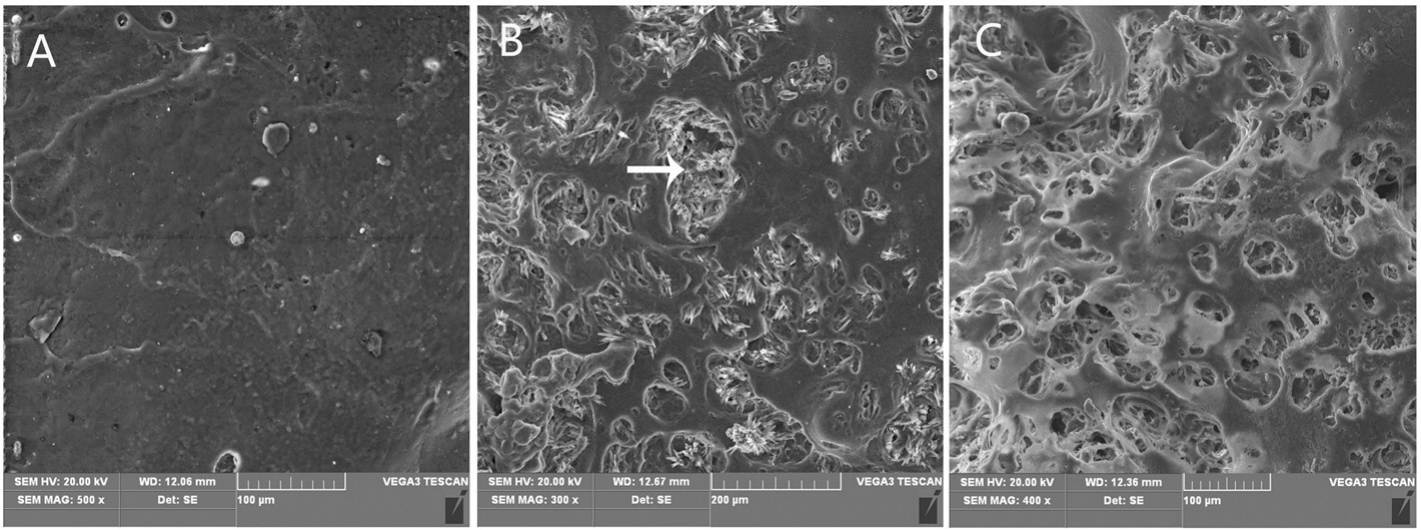
**Observation of the implant under microscope. A**: Microstructure of barrier layer (500×); **B**: Microstructure of the drug release area; the white arrows shows the INH (300×); **C**: Microstructure of the tablet after 30 days of drug release (400×).

The PLLA implant was observed under microscope and the result was shown in Figure 4. After post printing processing, the surface of the barrier layer was compact and only a few holes could be found. No INH could be observed on the barrier layer. There were many micropores with diameters around 50-100 um which were uniformly distributed in the drug release area. INH particles were contained in the micropore, embeded into the PLLA and distributed in nested way.

After 30 days of drug release, the INH in the drug release system was dissolved into the medium and many micropores were left on the surface of the implant. The pore size was larger than before.

### Result of the direct contact test

**Figure 5:**
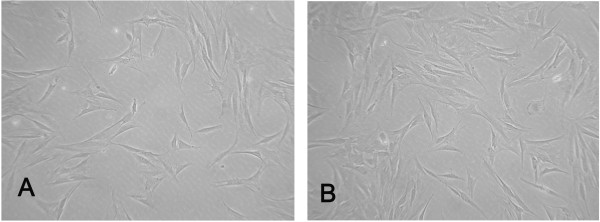
**Cells grew around the tablet. A**: The cellular morphology after 24 hours of incubation; **B**: The cellular morphology after 72 hours of incubation.

INH-PLLA implant and rat mesenchymal stem cells were co-incubated for 3 days. The cells grew well and attached. The number of cells increased from the first day to the third as shown in Figure 5. No abnormal or metamorphic cells were found around the implant, no reaction zone was discovered and the cells grew near the implant. There were no obvious differences beween the experimental groups and control groups in cell morphology, number and distribution. The cytotoxicity graded 0.

### MTT cytotoxicity test

The result of the cytotoxicity test is shown in Table 3. OD values between the experimental and control groups at 3 time points showed no significant difference. The RGR% in 24 h, 48 h and 72 h was 103%, 96% and 96%, respectively.

**Table 3:** MTT data of the PLLA implant and the statistical analysis results

**Group**	**OD Value**		
	**24 h**	**48 h**	**72 h**
**Experimental group (n = 5)**	0.696 ± 0.022	0.678 ± 0.035	0.680 ± 0.028
**Control group (n = 5)**	0.675 ± 0.031	0.701 ± 0.029	0.711 ± 0.023
**t**	1.24	1.13	1.91
**P**	>0.05	>0.05	>0.05

### Result of the in vitro drug release experiment

**Figure 6:**
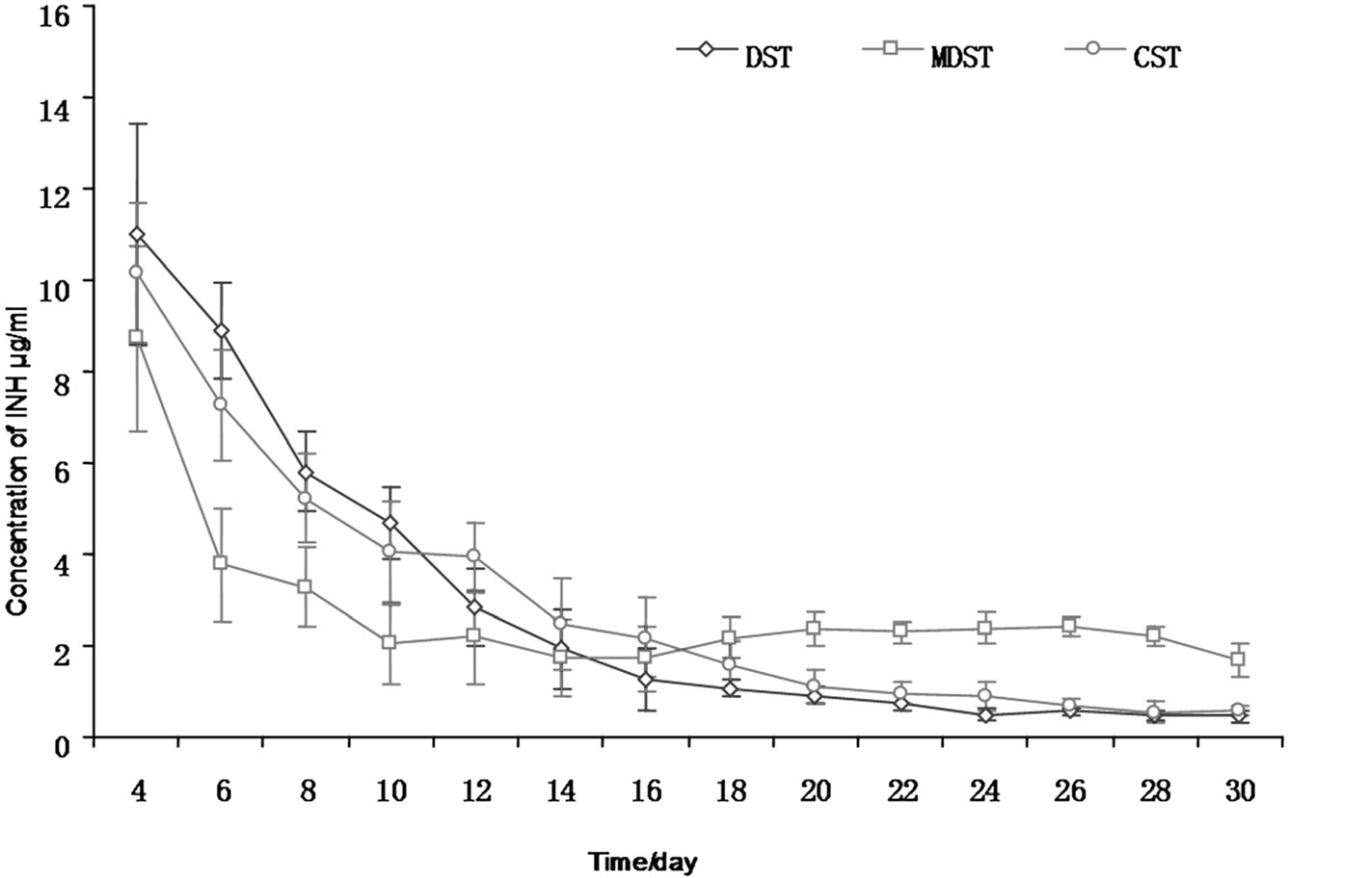
**INH released from the INH-PLLA implant fabricated by 3D printing technique in vitro.** Data shown as mean ± S.D., n = 5.

All of the three drug delivery systems showed initial burst release. The INH concentration of the MDST, DST and CST was 23.52 ± 2.49 μg/ml, 37.86 ± 3.28 μg/ml and 31.47 ± 3.51 μg/ml respectively, after two days of release. The concentration of INH decreased gradually as shown in Figure 6. The amount of the drug released from MDST became stable and fluctuated slightly from the 6th day onwards. The drug release rate of DST and CST both decreased gradually,but the rate of release in DST decreased faster compared to CST.

## Discussion

The rapid prototyping technique originated from the United States in the 1980s. The major characteristic of this technique was in the method of manufacture. Materials were piled up and combined layer by layer under the control of a computer, which completed the manufacture of the product. This technique did not rely on molds. Many types of rapid prototyping techniques had been developed ever since. 3DP technique attracted a lot of attention since it was proposed by Sachs etc. It could manufacture products with complex shapes and intricate internal structures. It also attracted extensive attention in relation to its application in the medical field. Tissue engineering scaffolds with unique structure could be prepared with the use of 3DP machines [12]. It also could make bone fracture models with 3D CT data for the doctor’s reference before operation, and also make individualized implants to fix bone deficits [13].

Three dimensional printing technique consisted of two major elements, which were binding liquid and matrix. The binding liquid and matrix were selected according to the requirement of the experiment. In this study, we used biodegradable PLLA as the matrix and the organic solvent with INH as the binding liquid [10,14]. The experimental results showed that slow release implants were obtained using the above materials which had excellent binding structures without disintegration. After post printing processing, slow release implant regained fine appearance and hardness without dropping any powders from the surface of the implant. PLLA was an ideal material to prepare biodegradable slow release implant. Observing the microstructure of the implant, we could find that the PLLA powder fused excellently after resolidification and packaged the INH evenly in a “nest shaped” way. On the surface of barrier layer, no INH crystallization could be found. The barrier layer blocked the INH release of MDST.

Local antibiotic drug delivery devices such as drug loading bone cement, bone screw and bone cement bradde chain played an important role in the prevention and treatment of bone infection [15-18]. The main point of those studies was to fabricate drug delivery devices that could release drugs steadily for long time in vivo. The drug release rate was related to its water solubility. Drug with higher water solubility was much easier to dissolve through the surface of the drug delivery device [19]. The INH used in this study had high water solubility. It dissolved gradually from the micropores on the surface of PLLA implant for 30 days. At the end of the experiment, the INH in the micropores of PLLA had disappeared, when observed under microscope.

In addition, the structure of the implant influenced the drug release pattern. In the CST, the drug release area decreased as the volume of the implant shrunk. While in the DST, the release area in the center of the implant enlarged after corrosion. This partially compensated for the loss in area of the outer surface. When comparing the drug release pattern of three different shaped implants, we could find that all three shapes of implant showed initial burst release, which may be related to the surface diffusion mechanism [20]. However, the drug release rate became stable after 4 days. A more stable drug release pattern could be found in MDST compared to the other two implants. The possible mechanism was that the porosity of the implant influenced the INH release. There were many micropores distributed in nest shaped way in the outer and inner surface of the MDST. The INH was mainly released from the micropore of the drug release area. No micropores could be observed on the barrier layers which blocked the release of INH. In the MDST structure, the release area could be well compensated due to the effect of the barrier layers after the center of the implant enlarged and maintained a constant surface area for drug release. However, there was no such complementary effect in CST and the drug release area decreased as the experiment progressed, which resulted in the decrease of the drug release rate. DST, which had the largest initial drug release surface area, showed the fastest initial drug release rate, and the drug release rate also descended at a faster rate as compared to the other two tablets.

The drug loading ability was restricted by the total volume of the implant. Thus the best loading drug should be effective in low concentrations with small total drug amount in need. INH was an ideal drug for local drug delivery with a minimal effective concentration of INH of 0.025 ~ 0.05 μg/ml. In the present research, the INH concentration of the three implants was higher than the effective bacteriostasis concentration within 30 days. The MDST tablet had the highest concentration with 30 days, as the drug release rate in MDST was relatively slow when compared to the other two tablets. This finding suggested that the MDST was a useful delivery vehicle, which could control the release of drug over a prolonged period of time while keeping the concentration of the drug relatively stable.

Clinical application of biomaterial should meet certain biological evaluation criterion. ISO10993 which was issued by the international organization for standardization was the most authoritative and widely used evaluation criterion. In this study, we conducted the in vitro cytotoxicity test to analyze the biocompatibility of the INH-PLLA implant. The results of direct contact test demonstrated that the INH-PLLA implant could coexist well with cells in vitro. There was no reaction region in the contact surface of the cell and the implant, cells grew well and distributed evenly around the implant, and the cytotoxicity still graded 0 after 3 days of co-incubation. The MTT cytotoxicity test showed that the OD value of the experimental group and the control group did not have a significant difference. RGR% in all groups was over 96%, which was higher than 70% as required by the ISO10993. The outcome mentioned above initially demonstrated that the implant did not have any obvious cytotoxicity.

Embolic arteritis commonly occurs around the focus of bone and joint tuberculosis. Poor or no blood supply in the focus resulted in the inability of the antitubercular agent to reach the focus after systemic drug delivery. While in using local antitubercular agent delivery, drugs were directly affected on the focus and high local drug concentration could be obtained with low total dosage and less systemic side effects. Drug release pattern of MDST could be divided into two phases which were the initial burst release and the later stable release. In the burst release phase, high dosage of INH could kill the mycobacterium tuberculosis in the focus post-operation, and the stable release phase which lasted over 30 days guaranteed the sustained bacteriostasis and consolidated curative effectiveness. This would provide a new method for the topical drug delivery of antituberculosis agent

## Conclusions

3DP was a reliable technique to fabricate complicated implants. The drug was evenly distributed in the implant. Among the three different structures of tablets, MDST showed the most stable drug release and therefore was an ideal drug delivery system for the antibiotic implant. The INH-PLLA implant met the biocompatibility requirements. The drug concentration released from the implant was still higher than the minimal effective concentration, even after 30 days of drug release. The multilayer donut-shaped tablet provided a new method of stable release for the topical application of the antibiotic.

## Abbreviations

CST: Columnar-shaped tablet; DST: Doughnut-shaped tablet; MDST: Multilayer doughnut-shaped tablet; PLLA: Poly L lactic acid.

## Competing interests

The authors declare that they have no conflict of interests related to this work.

## Authors’ contributions

QZ had full access to all of the data in the study and takes responsibility for the integrity of the data and the accuracy of the data analysis. Conception and design: GW, WW, QZ. Analysis and interpretation of the data: GW, JL, JZ. Drafting of the article: GW, ZH, QZ. Critical revision of the article for important intellectual content: GW, QZ. Final approval of the article:GW, WW, QZ, JL. Statistical analysis: GW, WW, JZ, ZH. Administrative, technical, and logistic support: WW. Collection and assembly of data: GW, QZ, JL. All authors read and approved the final manuscript of this article.
